# Hyperhomocysteinemia promotes lipid deposition in skeletal muscle

**DOI:** 10.3389/fneur.2026.1861632

**Published:** 2026-06-16

**Authors:** Menghan Su, Jiaqi Jiao, Junsen Zhao, Jing Ma, Huiqiu Zhang, Qiyun Liu, Juan Wang, Dan Liu, Qi Wen, Jianli Wang, Xueli Chang, Junhong Guo, Wei Zhang

**Affiliations:** 1 Department of Neurology, First Hospital, Shanxi Medical University, Taiyuan, China; 2 Research Center for Neurological Diseases, Shanxi Medical University, Taiyuan, China; 3 First Clinical Medical College, Shanxi Medical University, Taiyuan, China

**Keywords:** ACC2, C2C12 myotubes, CPT1 enzyme activity, hyperhomocysteinemia, lipid deposition, malonyl-CoA

## Abstract

**Background:**

Lipid storage myopathy (LSM) is characterized by abnormal lipid accumulation in skeletal muscle. Emerging evidence suggests that environmental factors, including the use of antidepressants such as sertraline, may trigger LSM. Given the established link between hyperhomocysteinemia (HHcy) and disrupted lipid metabolism, we investigated its potential role in skeletal muscle lipid deposition.

**Methods:**

We enrolled six patients with HHcy undergoing muscle biopsy and explored their clinical and pathological characteristics of skeletal muscle. The mechanistic link was explored in muscle tissues from patients through transcriptomic profiling, quantitative real-time polymerase chain reaction (qRT-PCR), western blotting, and enzymatic assays, and validated in C2C12 myotubes.

**Results:**

Four of the six patients presented with clinical myopathic manifestations, including progressive muscle weakness and exercise intolerance, which resolved completely after B-vitamin supplementation, while abnormal skeletal muscle lipid deposition was observed in all six patients. Transcriptome and qRT-PCR analyses demonstrated a significant upregulation of the acetyl-CoA carboxylase β (*ACACB*) gene (*p* < 0.001), which encodes acetyl-CoA carboxylase 2 (ACC2), in the muscle tissues from patients. Furthermore, ACC2 protein expression was markedly elevated (*p* < 0.01), thereby raising cellular malonyl-CoA levels (*p* < 0.01). This metabolite potently inhibits carnitine palmitoyltransferase 1 (CPT1), impairing fatty acid oxidative metabolism in skeletal muscle. The key molecular cascade involving *ACACB* upregulation and subsequent CPT1 inhibition (*p* < 0.05), was further verified in C2C12 myotubes.

**Conclusion:**

This study indicates that HHcy is closely associated with abnormal skeletal muscle lipid deposition. HHcy may correlate with increased ACC2 expression, which elevates malonyl-CoA levels. This, in turn, suppresses CPT1 activity and facilitates abnormal lipid accumulation in skeletal muscle.

## 1 Introduction

Lipid storage myopathy (LSM) arises from defects in intracellular triglyceride (TG) catabolism, long-chain fatty acid and carnitine transport, or fatty acid β-oxidation, and is pathologically defined by lipid accumulation within muscle fibers (1). Affected individuals typically present with muscle weakness, exercise intolerance, myalgia, rhabdomyolysis, or metabolic crisis (2). LSM is generally recognized as a group of Mendelian disorders caused by genetic variants in genes encoding enzymes essential for muscle fatty acid metabolism, such as primary carnitine deficiency (PCD), multiple acyl-coenzyme A dehydrogenase deficiency (MADD), and neutral lipid storage diseases with ichthyosis (NLSD-I), neutral lipid storage disease with myopathy (NLSD-M) and carnitine palmitoyltransferase (CPT) deficiency (3). Two recent independent reports indicate that sertraline treatment can induce MADD, suggesting environmental factors may also contribute to LSM (2, 4).

Hyperhomocysteinemia (HHcy), commonly defined by a serum total homocysteine (Hcy) (tHcy) concentration exceeding 15 μmol/L, is a well-established independent risk factor for coronary atherosclerosis, dementia, and non-alcoholic fatty liver disease. HHcy is also associated with a broad spectrum of other chronic disorders, including cardiovascular, neurological and metabolic disease (5–10). Importantly, emerging evidence highlights a connection between HHcy and systemic lipid metabolic disruption. Patients with HHcy consistently display elevated serum TG concentrations (10), and interventions that reduce Hcy levels ameliorate hepatic steatosis in rodent models (11, 12). At the cellular level, Hcy promotes abnormal lipid accumulation in foam cells during atherosclerotic plaque development (13) and suppresses lipolysis in adipocytes, resulting in intracellular TG accumulation (14).

Despite compelling evidence linking HHcy to lipid metabolic disturbances across various tissues, its potential role in skeletal muscle lipid metabolism and related lipid deposition remains unexplored. In this study, we aimed to investigate the clinical and skeletal muscular pathological features of six HHcy patients with confirmed intramyocellular lipid deposition, including one patient with LSM and five patients with subacute combined degeneration (SCD) of the spinal cord. We further sought to elucidate the pathogenic mechanism responsible for HHcy-induced intramyocellular lipid accumulation.

## 2 Methods

### 2.1 Subjects

We retrospectively reviewed patients from the Neuromuscular Unit of the First Hospital at Shanxi Medical University between 2020 and 2025. The study enrolled patients with HHcy who underwent skeletal muscle biopsy. We excluded individuals with genetic etiologies or a relevant family history. Medical records provided patient characteristics, comorbidities, clinical symptoms, physical signs, and diagnostic investigations. The Institutional Ethics Committee of the First Hospital of Shanxi Medical University approved this study (Approval NO. KYLL-2025-252), which adhered to the ethical principles of the 1964 Declaration of Helsinki and its subsequent amendments.

### 2.2 Muscle biopsy and histopathological analysis

Skeletal muscle biopsies were processed for histopathological examination using a standard panel of histological and histochemical stains, as previously described (15). We visualized lipid accumulations with oil red O (ORO) staining. For fluorescent labeling of lipid droplets, we also employed BODIPY™ 493/503 (Invitrogen). Briefly, sections were washed three times with phosphate-buffered saline (PBS) and fixed in 4% (w/v) paraformaldehyde for 10 min at room temperature. After two additional PBS washes, the cryosections were stained with 3.8 μmol/L BODIPY™ 493/503 for 20 min at room temperature. The samples were then washed three times with PBS and counterstained with DAPI for 1.5 min. Following a final wash, sections were mounted on glass slides using Antifade Mounting Medium (P0126, Beyotime) and sealed. An Olympus BX53 fluorescence microscope (Olympus) captured the images. For ultrastructural analysis, we processed muscle specimens according to a previously established protocol (16). Control skeletal muscle tissues consisted of biopsy specimens from one subject without LSM.

### 2.3 Transcriptomic analysis

Total RNA was extracted from frozen human skeletal muscle tissues. RNA quality and integrity were assessed using the LabChip Bioanalyzer and the 5,300 Fragment Analyzer automated electrophoresis system. RNA-seq libraries were constructed using the Yeasen RNA Library Preparation Kit (Cat. 12340ES97, China) and sequenced on the Illumina NovaSeq 6000 platform by LC Bio Technology (Hangzhou, China). Raw sequencing reads were subjected to quality assessment using FastQC. Adapter sequences and low-quality reads were removed to obtain clean reads. Clean reads were aligned to the human reference genome GRCh38 using HISAT2. Transcript assembly and gene expression quantification were performed with StringTie. Gene expression levels were normalized as FPKM (Fragments Per Kilobase of transcript per Million mapped reads). Transcriptome coverage distribution and genome mapping rates were evaluated for each sample. Principal component analysis (PCA) and sample correlation analyses were conducted to assess sample similarity, clustering patterns, and potential batch effects. No obvious batch effect was observed among samples, therefore, no additional batch correction was performed. Differentially expressed genes (DEGs) were screened with the threshold of |log2(fold change)| ≥ 1 and *q*-value <0.05. The volcano plot of DEGs was generated using the LC Bio Cloud Platform.

### 2.4 Cell culture and differentiation

C2C12 mouse myoblasts were cultured in growth medium (Dulbecco’s Modified Eagle Medium (DMEM) supplemented with 10% fetal bovine serum (FBS) and 1% penicillin–streptomycin) at 37 °C in a 5% CO₂ atmosphere. To induce myogenic differentiation, the growth medium was replaced with differentiation medium (DMEM containing 2% horse serum and 1% penicillin–streptomycin) once cells reached 80% confluence. The differentiation medium was refreshed every 24 h. To evaluate the effects of HHcy, we added Hcy (H4628, Sigma-Aldrich) at concentrations of 0 μmol/L, 25 μmol/L, or 250 μmol/L starting on day 2 of differentiation, with fresh medium supplied daily. After 5 days of treatment (on day 6 post-differentiation), samples were collected for analysis.

### 2.5 BODIPY staining of C2C12 myotubes

On day 5 of differentiation, we introduced 200 μmol/L palmitic acid (607489, Sigma) into the differentiation medium to induce lipid accumulation. The cells were subsequently incubated for 24 h. Finally, the myotubes were stained with BODIPY™ 493/503.

### 2.6 Quantitative real-time polymerase chain reaction (qRT-PCR)

Total RNA was isolated from patient muscle tissues and C2C12 myotubes using an RNA isolation kit (Vazyme, China). RNA concentration, purity, and integrity were assessed by measuring 260/230 and 260/280 ratios on a NanoDrop spectrophotometer; 500 ng of RNA was used for cDNA synthesis. We determined gene expression levels via qRT-PCR, with primer sequences provided in Supplementary Table S1. Data were normalized to β-actin expression.

### 2.7 TG quantification

We quantified intramuscular and intracellular TG content using a commercial TG assay kit (E1013-105, Applygen Technologies Inc., Beijing, China) following the manufacturer’s instructions (17).

### 2.8 Assessment of malonyl-CoA content

Malonyl-CoA concentrations in the patient muscle tissues and treated C2C12 myotubes were measured with a commercial enzyme-linked immunosorbent assay (ELISA) kit (MM-51489H1, Jiangsu Meimian Industrial Co., Ltd., China) according to the manufacturer’s instructions.

### 2.9 CPT1 enzyme activity assay

We determined CPT1 enzyme activity in muscle tissues and cell homogenates using a commercial assay kit (PMK1153, Pumoke Biotechnology Co., Ltd., Wuhan, China).

### 2.10 Western blot analysis of acetyl-CoA carboxylase (ACC), phospho-ACC (p-ACC), acetyl-CoA carboxylase 2 (ACC2), and carnitine palmitoyltransferase 1B (CPT1B) expression levels

Proteins were extracted from patient muscle tissues and treated C2C12 myotubes and quantified with a BCA protein assay kit (BOSTER, China). Equal amounts of protein were separated by sodium dodecyl sulfate-polyacrylamide gel electrophoresis (SDS-PAGE) and transferred onto polyvinylidene fluoride (PVDF) membranes. The membranes were blocked with 5% BSA for 3 h at room temperature and then incubated overnight at 4 °C with the following primary antibodies: ACC (1:1000, CY5575, Abways), p-ACC (Ser79) (1:1000, D7D11, Cell Signaling Technology), ACC2 (1:1000, YT7481, Immunoway) and CPT1B (1:1000, E6M5M, Cell Signaling Technology). The ACC antibody recognizes both ACC1 and ACC2 isoforms, and the p-ACC (Ser79) antibody detects the phosphorylated forms of both ACC1 and ACC2. The ACC2 antibody is highly specific to ACC2 and exhibits no cross-reactivity with ACC1. After washing, the membranes were incubated with horseradish peroxidase-conjugated secondary antibodies for 1.5 h at room temperature. Protein bands were visualized and analyzed using a ChemiDoc MP imaging system.

### 2.11 Statistical analysis

Statistical analyses were conducted with GraphPad Prism 8 (GraphPad Prism, Inc., San Diego, CA, USA). Normally distributed data were assessed by one-way analysis of variance (ANOVA), followed by Tukey’s test for *post hoc* pairwise comparisons, and results are reported as means ± SEMs. For data that did not follow a normal distribution, the Kruskal-Wallis test was employed, followed by Dunn’s multiple comparison test for subsequent pairwise comparisons, and results are presented as median and interquartile range (IQR). A *p* < 0.05 was considered statistically significant.

## 3 Results

### 3.1 Clinical features

This study enrolled six patients with HHcy: one diagnosed with LSM (Patient 2) and five with SCD (Patients 1, 3–6). Table 1 summarizes their clinical characteristics. Comorbidities included macrocytic anemia (*n* = 4), normocytic anemia (*n* = 1), depression (*n* = 1), hypothyroidism (*n* = 1), and Hashimoto’s thyroiditis (*n* = 1). None of the patients received sertraline, and five reported a low-meat diet. Progressive muscle weakness and exercise intolerance developed in four patients, representing the initial symptoms in two. Syncope occurred in two patients, and one experienced a single seizure. Physical examination revealed decreased proximal lower extremity muscle strength in two patients. Laboratory investigations identified vitamin B_12_ deficiency (*n* = 5; reference range: 133–675 pmol/L), folate deficiency (*n* = 1; reference range: >10 nmol/L), and elevated Hcy levels (*n* = 6; reference range: 0–15 μmol/L). One patient exhibited increased creatine kinase (CK: female 40–200 U/L; male 50–310 U/L), while blood acylcarnitine profiles were normal in all cases. Whole-exome sequencing in Patients 1 and 2 detected no clinically relevant variants. Treatment with mecobalamin and folic acid resulted in complete resolution of muscle weakness and exercise intolerance within 2 months.

**Table 1:** Detailed clinical and laboratory findings.

Patient	1	2	3	4	5	6	All patients
Gender (male/female)	F	M	F	F	M	M	3/3
Age at onset	22	14	45	75	53	63	Mean 45.33
Age at diagnosis	23	17	46	76	54	63	Mean 46.50
SCD	Yes	No	Yes	Yes	Yes	Yes	5/6
Megaloblastic anemia	Yes	No	No	Yes	Yes	Yes	4/6
Other comorbidities	Depression			Hypothyroidism		Hashimoto’s thyroiditis	
Low-meat dietary intake	Yes	No	Yes	Yes	Yes	Yes	5/6
Symptoms							
Muscle weakness	Yes	Yes	Yes	Yes	No	No	4/6
Exercise intolerance	Yes	Yes	Yes	Yes	No	No	4/6
Myalgia	No	No	No	No	No	No	0/6
Rhabdomyolysis	No	No	No	No	No	No	0/6
Syncope	Yes	Yes	No	No	No	No	2/6
Seizure	No	Yes	No	No	No	No	1/6
Signs							
Muscle strength (MRC)							
Proximal UL	5	5	5	5	5	5	
Distal UL	5	5	5	5	5	5	
Proximal LL	3	4	5	5	5	5	
Distal LL	5	5	5	5	5	5	
Sensory findings	Yes	No	Yes	Yes	Yes	Yes	5/6
Abnormal tendon reflexes	No	No	No	Yes	Yes	Yes	3/6
Romberg sign	+	−	+	+	+	+	5/6
Babinski sign	+	−	−	+	+	−	3/6
Serum B12, pmol/L	<3.7 ↓	109 ↓	44 ↓	134	54 ↓	<37 ↓	Mean 63.62
Serum folate, nmol/L	17.9	9.5 ↓	54.15	23.3	54.15	41.9	Mean 33.48
Serum Hcy, μmol/L	170.4 ↑	59.7 ↑	126.2 ↑	80.5 ↑	126.1 ↑	112.1 ↑	Mean 112.50
MCV, fL	112 ↑	92.6	91.6	117.4 ↑	106.6 ↑	112.2 ↑	Mean 105.40
Serum creatine kinase, U/L	187	115	837↑	84	95	59	Mean 229.50
Elevated acylcarnitines in blood	No	No	No	No	No	No	0/6
Needle EMG	NT	Normal	Normal	Normal	Normal	Normal	5/5
Lipid storage	Yes	Yes	Yes	Yes	Yes	Yes	6/6
Treatment regimen	Mecobalamin injection 500–1,000 μg daily and folic acid tablet 5 mg daily						6/6
Muscle weakness resolution post-treatment	Yes	Yes	Yes	Yes	/	/	4/4

EMG, electromyography; Hcy, homocysteine; LL, lower limb; MCV, mean corpuscular volume; MRC, Medical Research Council; NT, not tested; SCD, subacute combined degeneration; UL, upper limb.

### 3.2 Muscle biopsy and histopathological analysis

**Figure 1:**
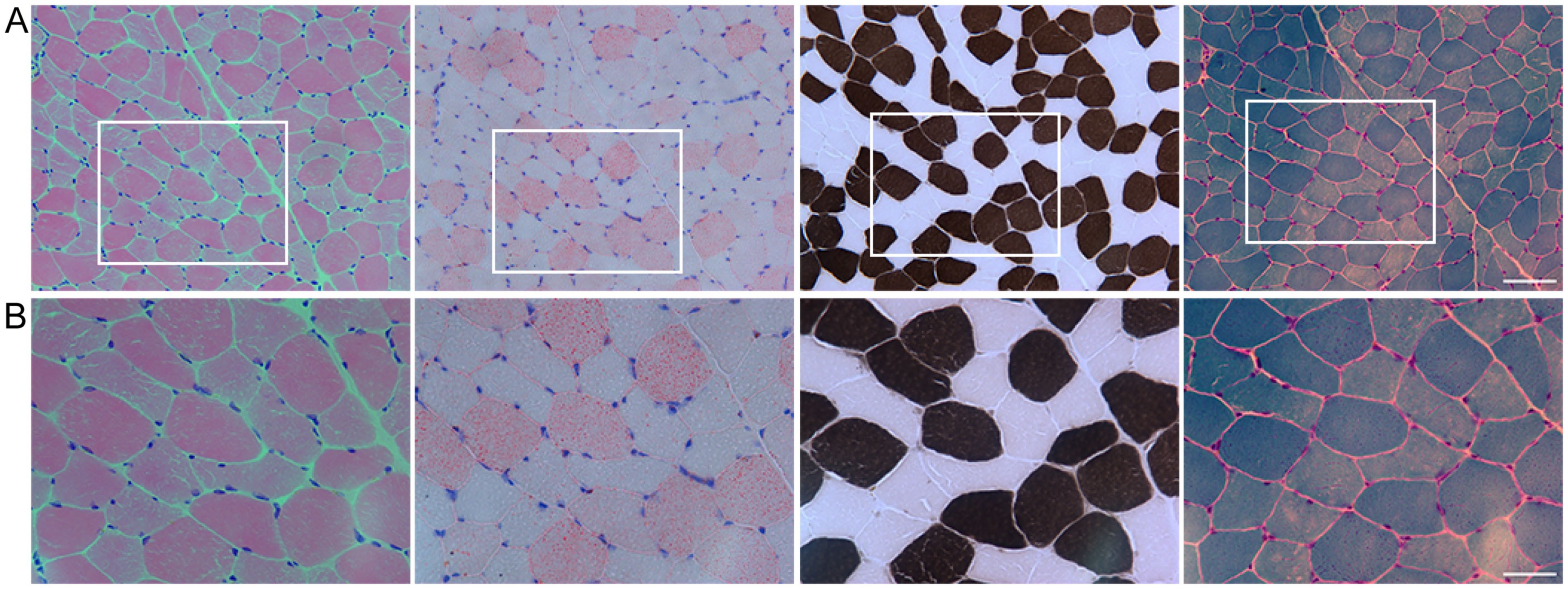
Muscular histopathological changes in Patient 4. **(A)** From left to right, panels show Hematoxylin and eosin (HE) staining, Oil Red O (ORO) staining, ATPase staining at pH 4.4, and modified Gomori trichrome (MGT) staining at 20x magnification. The HE staining revealed features of vacuolar myopathy, with blue nuclei and red cytoplasmic staining. ORO staining highlighted lipid deposition within muscle fibers as orange-red, while nuclei appear blue from counterstaining. Scale bar = 100 μm. **(B)** The corresponding 40× magnification of the boxed region from the 20x image is presented. From left to right, these panels display HE staining, ORO staining, ATPase staining at pH 4.4, and MGT staining at 40x magnification. Scale bar = 50 μm.

**Figure 2:**
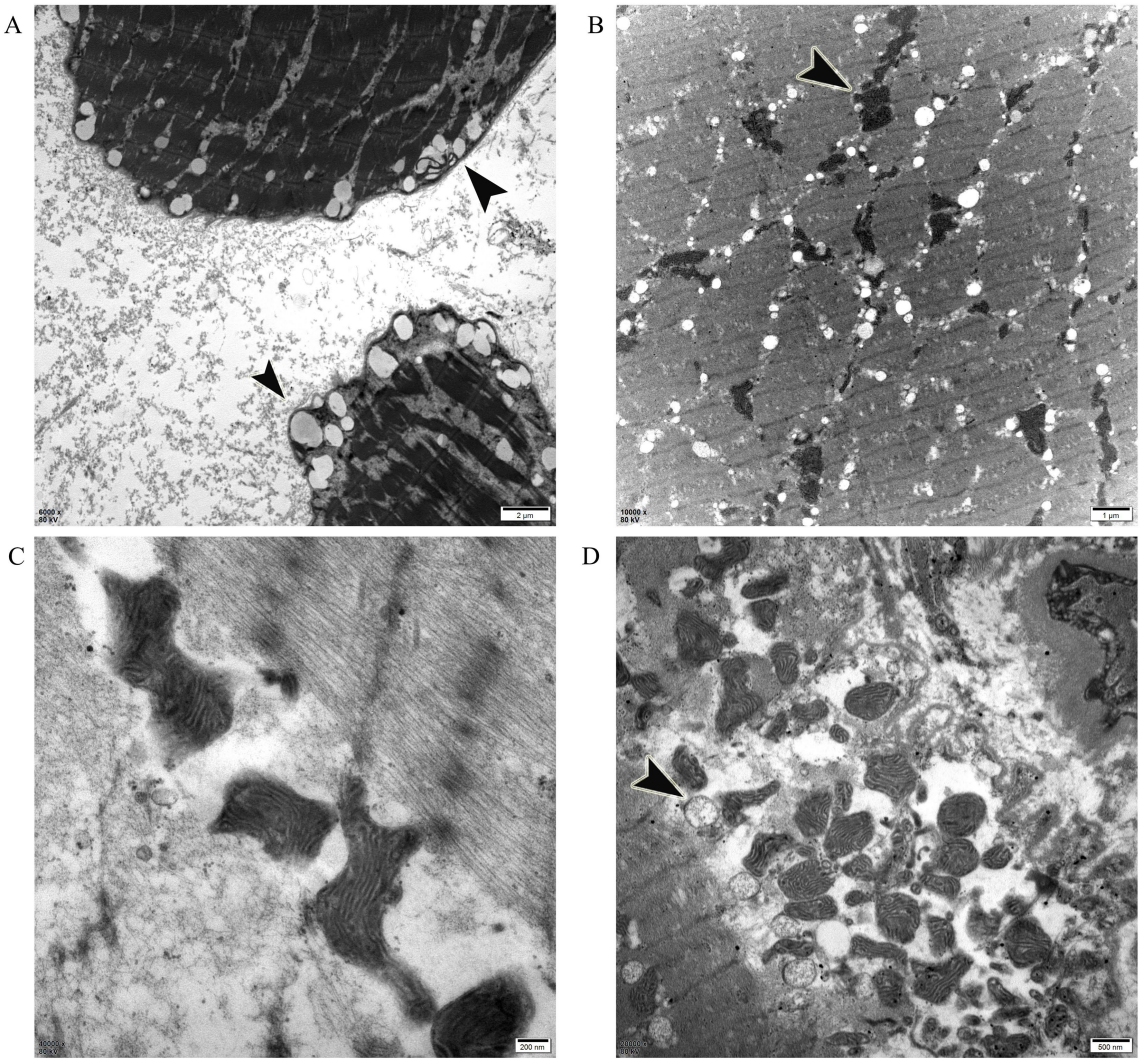
Electron micrograph of muscle in Patient 3. **(A)** Clusters of lipid droplets in the muscle fibers were seen in a subsarcolemmal region with increased size (arrowhead). **(B,C)** There were enlarged mitochondria with pleomorphic morphology. **(D)** An abnormally high density of mitochondria accompanied by mitochondrial vacuolar degeneration (arrowhead) was observed in this fiber.

Biopsies were obtained from the gastrocnemius (*n* = 1), quadriceps femoris (*n* = 4), and vastus lateralis (*n* = 1). All patients exhibited vacuolar myopathy with lipid storage (Figure 1). Lipid staining with ORO revealed mildly to moderately increased lipid accumulation, predominantly affecting type 1 muscle fibers. Modified Gomori trichrome, periodic acid-Schiff, NADH-tetrazolium reductase, cytochrome c oxidase, and succinate dehydrogenase stains showed no significant abnormalities. Electron microscopy performed in all six patients demonstrated lipid droplets clusters, mitochondrial proliferation, and vacuolar degeneration (Figure 2). Some patients exhibited enlarged mitochondria.

### 3.3 HHcy promoted lipid deposition in both muscle tissues from patients and C2C12 myotubes

**Figure 3:**
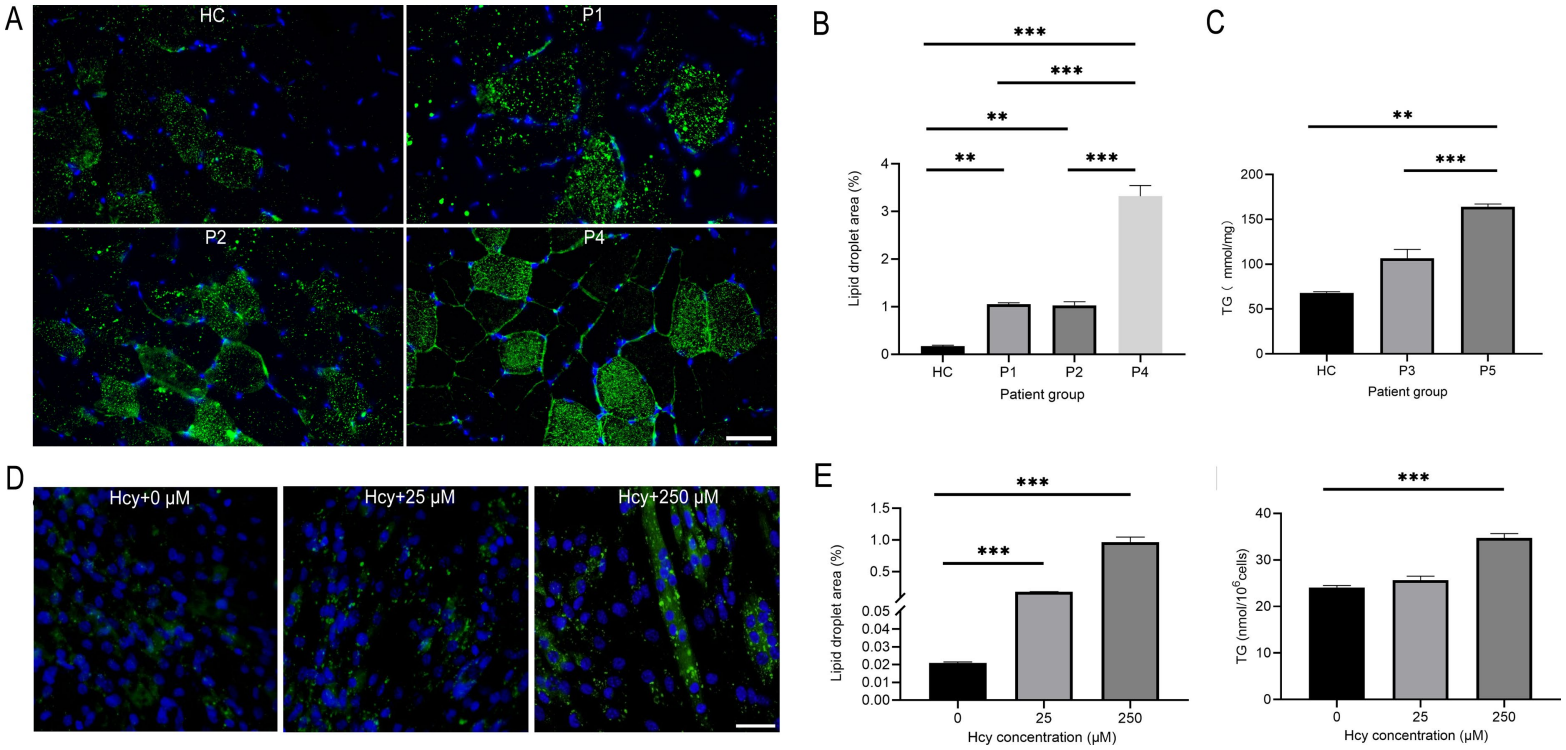
HHcy promoted lipid accumulation and increased TG content in skeletal muscle and C2C12 myotubes. **(A)** BODIPYTM 493/503 staining of patient muscle tissues from Patients 1, 2 and 4 showing increased lipid droplets compared to healthy control. Scale bar = 50 μm. **(B)** Quantification of lipid deposition from BODIPYTM 493/503 staining. **(C)** TG content in muscle tissues from Patients 3 and 5. Data are presented as mean ± SEM (*p < 0.05, **p < 0.01, ***p < 0.001 compared with HC). **(D)** BODIPYTM 493/503 staining of cells in different intervention groups. BODIPYTM 493/503 staining demonstrated that cells exposed to 25 μM or 250 μM Hcy exhibited a progressively greater accumulation of lipid droplets compared to those incubated with 0 μM Hcy. Scale bar = 50 μm. **(E)** Quantification of BODIPY fluorescence intensity. Data are mean ± SEM (^*^p < 0.05, ^**^p < 0.01, ^***^p < 0.001 compared with Hcy + 0 μM group). **(F)** Intracellular TG content in Hcy-treated myotubes. Data are mean ± SEM (^*^p < 0.05, ^**^p < 0.01, ^***^p < 0.001 compared with Hcy + 0 μM group). HC = healthy control, P1 = Patient 1, P2 = Patient 2, P3 = Patient 3, P4 = Patient 4, P5 = Patient 5, Hcy + 0 μM = differentiation medium, Hcy + 25 μM = differentiation medium + 25 μmol/L Hcy, Hcy + 250 μΜ = differentiation medium + 250 μmol/L Hcy.

BODIPY™ 493/503 staining revealed increased intramyofiber lipid droplets in Patients 1, 2, and 4 compared to the control (*p* < 0.01; Figures 3A,B). To validate these histological findings, we quantitatively assessed cytoplasmic TG levels in muscle tissues from Patients 3 and 5. The results demonstrated that muscle tissues from patients exhibited significantly higher TG content compared to the control (*p* < 0.01; Figure 3C). Cytological analysis further confirmed these observations. BODIPY™ 493/503 staining and TG content analysis in C2C12 myotubes demonstrated significantly increased lipid droplet accumulation following treatment with 25 μmol/L and 250 μmol/L of Hcy (*p* < 0.001; Figures 3D,E). Moreover, treatment with 250 μmol/L Hcy significantly increased intracellular TG levels (*p* < 0.001; Figure 3F).

### 3.4 HHcy increased acetyl-CoA carboxylase β *(ACACB)* mRNA levels and ACC2 protein abundance

**Figure 4:**
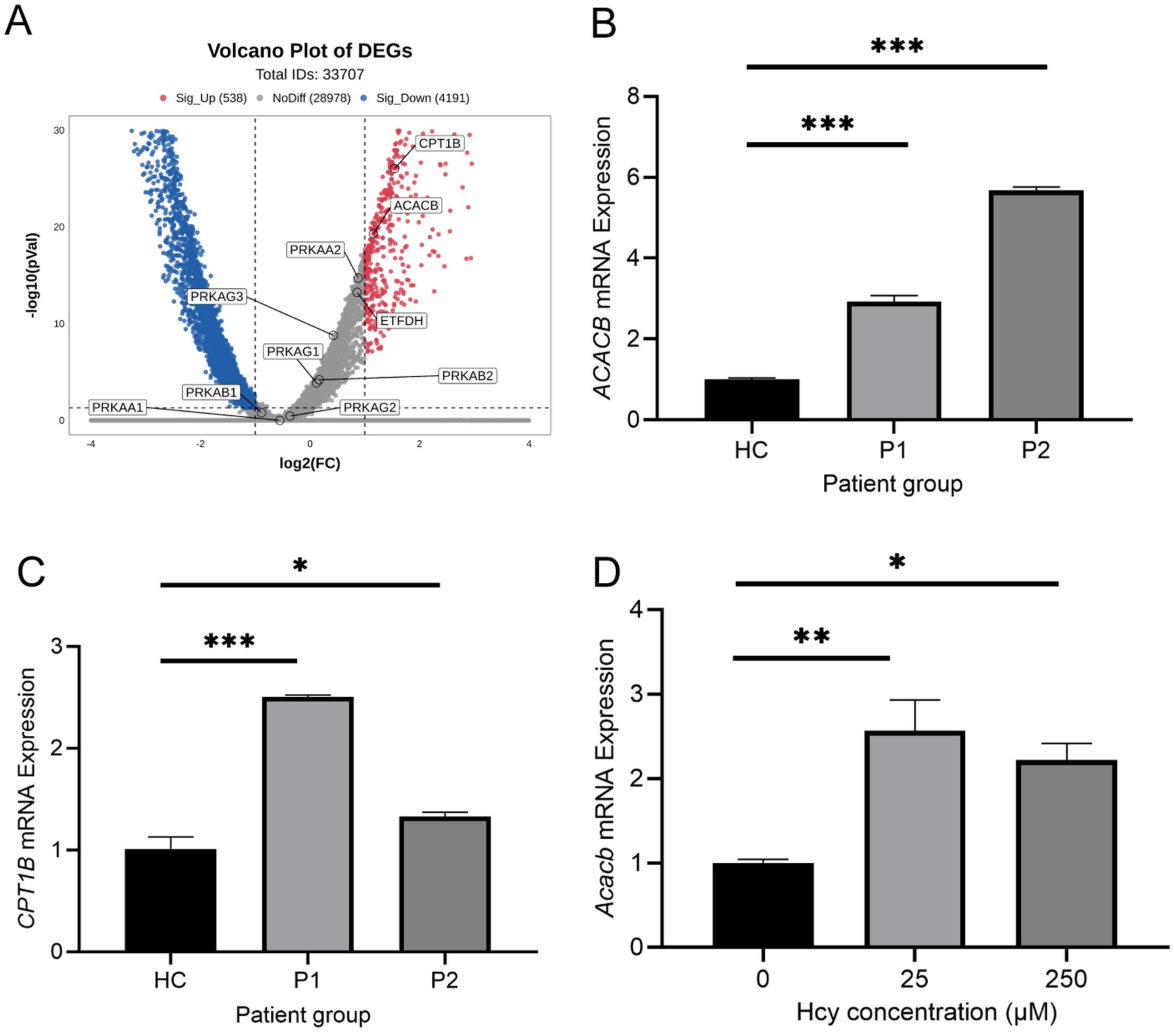
HHcy led to elevated ACACB and CPT1B mRNA expression. **(A)** Volcano diagram of DEGs in the muscle tissues between patients group compared with healthy control group. Red indicates up, blue indicates down. **(B,C)** ACACB and CPT1B mRNA levels in muscle tissues from Patients 1 and 2 compared with healthy control group. HC = healthy control, P1 = Patient 1, P2 = Patient 2 Data are mean ± SEM (^*^p < 0.05, ^**^p < 0.01, ^***^p < 0.001 compared with healthy control group). **(D)** Acacb mRNA levels in C2C12 myotubes treated with Hcy. Data are mean ± SEM (^*^p < 0.05, ^**^p < 0.01, ^***^p < 0.001 compared with Hcy + 0 μM group). Hcy + 0 μM = differentiation medium, Hcy + 25 μM = differentiation medium + 25 μmol/L Hcy, Hcy + 250 μΜ = differentiation medium + 250 μmol/L Hcy.

The above results demonstrated that HHcy promotes lipid deposition in both patients and Hcy-treated myotubes. To elucidate the underlying molecular mechanisms, we performed transcriptome analysis on muscle tissues from Patients 1 and 2 and a healthy control. The number of DEGs is illustrated in Figure 4A. Comparison of gene expression profiles between the patient group and the healthy control group revealed 4,729 significantly altered genes, with 538 genes up-regulated and 4,191 genes down-regulated. Considering the limited sample size, we selectively focused on key candidate genes for subsequent validation. Among these, transcriptional alterations in *ACACB* and *CPT1B* genes were particularly notable.

The *ACACB* gene encodes ACC2, which produces malonyl-CoA to inhibit CPT1B activity (18–22). ACC2 is inhibited by AMP-activated protein kinase (AMPK) via phosphorylation. Notably, the transcriptome analysis demonstrated significant upregulation of *ACACB* (fold change = 2.23, *p* < 0.001) and *CPT1B* (fold change = 2.92, *p* < 0.001) gene expression in muscle tissues, while the genes encoding AMPK showed no significant change (Figure 4A). We further validated the expression of *ACACB* and *CPT1B* genes by qRT-PCR. Our findings revealed that *ACACB* and *CPT1B* mRNA levels were significantly elevated in muscle tissues from patients (*p* < 0.05; Figures 4B,C). Subsequent experiments utilizing the myotube model confirmed that treatment with 25 μmol/L and 250 μmol/L Hcy resulted in a significant increase in *Acacb* mRNA levels (*p* < 0.05; Figure 4D).

**Figure 5:**
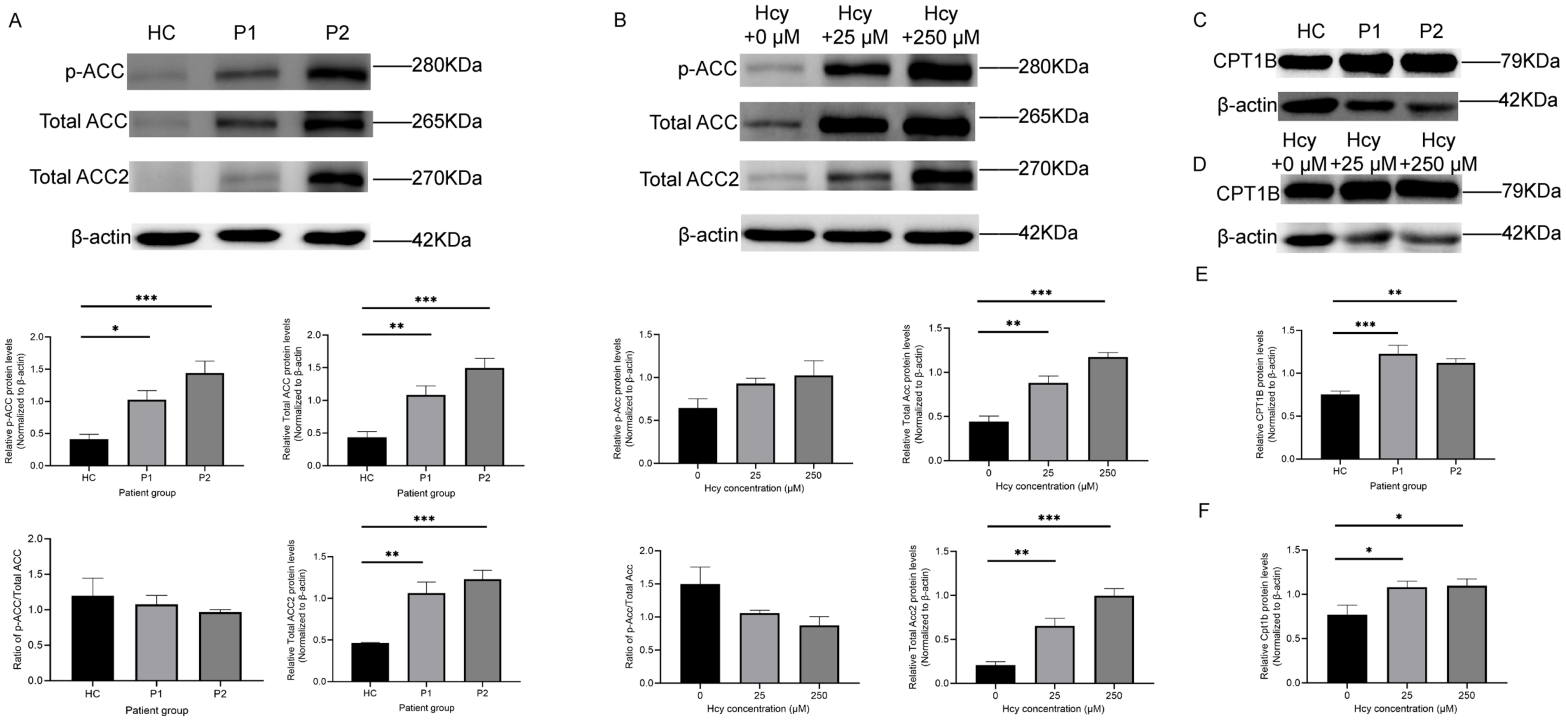
HHcy upregulated ACC and ACC2 protein expression without altering ACC phosphorylation. **(A)** The expression levels of p-ACC (Ser79), total ACC, and total ACC2 protein in patient muscle tissues. Data are mean ± SEM (^*^p < 0.05, ^**^p < 0.01, ^***^p < 0.001 compared with healthy control group). **(B)** The expression levels of p-Acc, Acc, and Acc2 in C2C12 myotubes after treatment with Hcy. Data are mean ± SEM (^*^p < 0.05, ^**^p < 0.01, ^***^p < 0.001 compared with the Hcy + 0 μM group). **(C,E)** The expression levels of CPT1B protein in patient muscle tissues. Data are mean ± SEM (^*^p < 0.05, ^**^p < 0.01, ^***^p < 0.001 compared with healthy control group). **(D,F)** The expression levels of Cpt1b in C2C12 myotubes after treatment with Hcy. Data are mean ± SEM (^*^p < 0.05, ^**^p < 0.01, ^***^p < 0.001 compared with the Hcy + 0 μM group). HC = healthy control, P1 = Patient 1, P2 = Patient 2, Hcy + 0 μM = differentiation medium, Hcy + 25 μM = differentiation medium + 25 μmol/L Hcy, Hcy + 250 μΜ = differentiation medium + 250 μmol/L Hcy.

Consistent with this transcriptional upregulation, total ACC2, total ACC, and CPT1B protein expression increased in muscle tissues from patients (*p* < 0.05; Figures 5A,C,E), whereas ACC phosphorylation levels remained unaltered (Figure 5A). These findings indicate that HHcy-associated lipid accumulation in muscle fibers is mediated by ACC2 protein upregulation rather than phosphorylation changes. Correspondingly, exposure to 25 μmol/L and 250 μmol/L Hcy markedly increased total Acc2, total Acc, and Cpt1b protein expression (*p* < 0.05; Figures 5B,D,F) without altering Acc phosphorylation status in C2C12 myotubes (Figure 5B).

### 3.5 HHcy elevated malonyl-CoA levels and suppressed CPT1 enzyme activity

**Figure 6:**
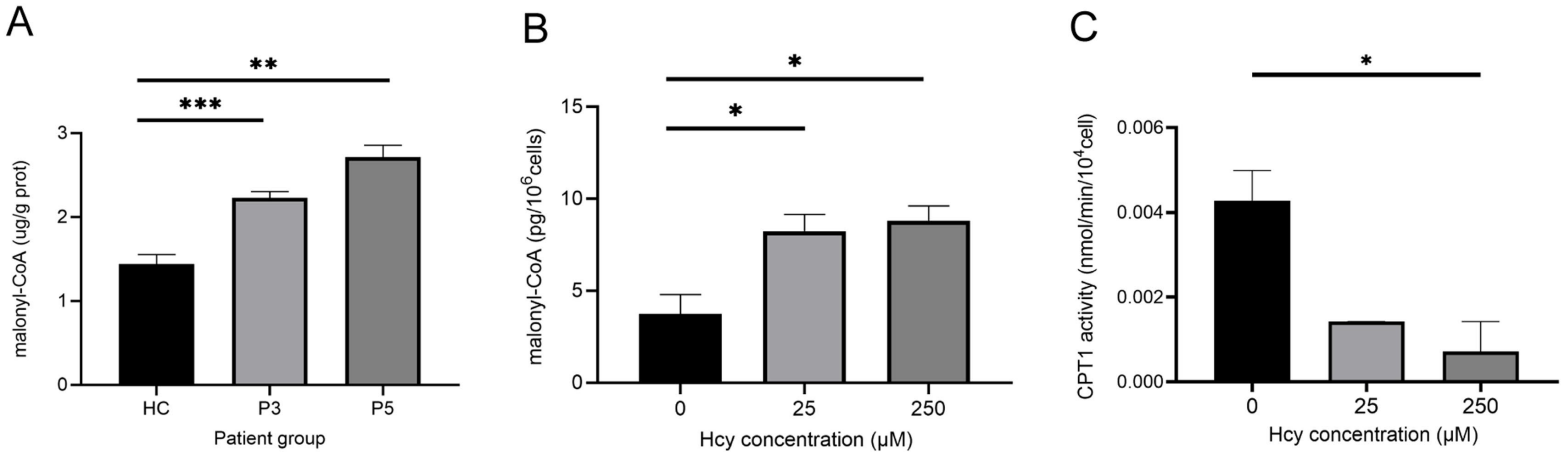
HHcy elevated malonyl-CoA levels and suppressed CPT1 enzyme activity. **(A)** The expression levels of malonyl-CoA in patient muscle tissues. Data are mean ± SEM (^*^p < 0.05, ^**^p < 0.01, ^***^p < 0.001 compared with healthy control group). HC = healthy control, P3 = Patient 3, P5 = Patient 5 **(B)** The expression levels of malonyl-CoA in C2C12 myotubes after treatment with Hcy. Data are mean ± SEM (^*^p < 0.05, ^**^p < 0.01, ^***^p < 0.001 compared with the Hcy + 0 μM group). Hcy + 0 μM = differentiation medium, Hcy + 25 μM = differentiation medium + 25 μmol/L Hcy, Hcy + 250 μΜ = differentiation medium + 250 μmol/L Hcy. **(C)** The activity of CPT1 enzyme in C2C12 myotubes after treatment with Hcy. Data are presented as median and interquartile range (IQR) (^*^p < 0.05, ^**^p < 0.01, ^***^p < 0.001 compared with the Hcy + 0 μM group). Hcy + 0 μM = differentiation medium, Hcy + 25 μM = differentiation medium + 25 μmol/L Hcy, Hcy + 250 μΜ = differentiation medium + 250 μmol/L Hcy.

To validate the functional consequences of ACC2 upregulation, we measured the levels of its product, malonyl-CoA. Muscle tissues from Patients 3 and 5 showed significantly increased malonyl-CoA (*p* < 0.01; Figure 6A). This finding was further corroborated in C2C12 myotubes, where treatment with 25 μmol/L and 250 μmol/L Hcy also markedly elevated malonyl-CoA levels compared to the control (*p* < 0.05; Figure 6B). As malonyl-CoA is a natural CPT1 inhibitor that decreases fatty acid β-oxidation and promotes lipid accumulation, we measured CPT1 activity in C2C12 myotubes. The results indicated a significant decrease in CPT1 activity in C2C12 myotubes treated with 250 μmol/L Hcy (*p* < 0.05; Figure 6C).

## 4 Discussion

Given the established association between HHcy and lipid metabolic disorders, together with the clinical observation that some HHcy patients present with exercise intolerance (23), we aimed to explore the potential relationship between HHcy and skeletal muscle lipid deposition. In this study, pathological examination revealed lipid deposition in muscle fibers of the six included patients with HHcy, and myotube cells treated with Hcy *in vitro*. We further explored the underlying mechanism and found that HHcy is correlated with the upregulation of *ACACB* expression. This is accompanied by an increase in malonyl-CoA levels, which may reduce CPT1 activity, ultimately suppress mitochondrial fatty acid *β*-oxidation and facilitate lipid accumulation in skeletal muscle.

Notably, LSM is a clinically, biochemically, and genetically heterogeneous disorder with diverse etiologies, which cannot be attributed to a single cause (24). Previous studies have identified four major subtypes with distinct and characteristic biomarkers: PCD presents with markedly decreased serum free carnitine and reduced acylcarnitines; NLSD-M exhibits lipid droplets in leukocytes and decreased acylcarnitines; riboflavin-responsive MADD (RR-MADD) shows elevated acylcarnitines and may present with glutaric aciduria; and CPT deficiency demonstrates normal carnitine levels with specifically increased long-chain acylcarnitines (25). In our study, whole-exome sequencing was performed in only two patients, and no pathogenic variants associated with classic LSM were identified. All patients exhibited normal blood acylcarnitine profiles and achieved complete remission of myopathic symptoms after B-vitamin supplementation, indicating that the lipid deposition observed is a reversible metabolic alteration induced by HHcy rather than primary inherited LSM. HHcy therefore serves as an associated or precipitating factor for the skeletal muscle lipid deposition observed in this study, rather than a primary genetic cause of classic LSM.

Muscle biopsies from all six patients in this study revealed abnormal intramyofiber lipid accumulation. Their clinical presentations, however, were heterogeneous: four patients exhibited progressive muscle weakness and exercise intolerance, whereas Patients 5 and 6 lacked overt muscular symptoms. The complete resolution of myopathic manifestations in all symptomatic individuals following Hcy normalization through B-vitamin supplementation suggests a close associative relationship between HHcy and skeletal muscle lipid deposition. The cases of Patients 5 and 6 carry important clinical implications, suggesting that HHcy-induced skeletal muscle toxicity may precede clinically recognizable symptoms.

Notably, five of the six patients also presented with vitamin B_12_ deficiency, raising the question of whether low vitamin B_12_ status contributes to intramuscular lipid deposition. However, in our *in vitro* experiments, myotubes cultured under vitamin B_12_-sufficient conditions still exhibited abnormal lipid metabolism after Hcy intervention, recapitulating the pathological features observed in clinical muscle specimens. Collectively, these findings indicate that HHcy is closely associated with intramuscular lipid deposition independent of vitamin B_12_ deficiency.

The association between HHcy and skeletal muscle lipid deposition remains underrecognized, mainly for two principal reasons. First, the muscle manifestations resulting from lipid deposition in skeletal muscle may be overshadowed by the more prominent neurological deficits of SCD. The latter also stems from vitamin B_12_ deficiency, a common cause of HHcy. In such cases, clinicians may attribute all neurological and muscular dysfunction to SCD alone, thereby overlooking a concomitant myopathic pathology. Second, the lipid deposition observed in muscle biopsies was mild to moderate. This relatively subtle histopathological finding, particularly in the context of normal or mildly elevated creatine kinase levels and the absence of severe motor deficits, may not justify a muscle biopsy or prompt an extensive metabolic workup, thereby contributing to underdiagnosis.

**Figure 7:**
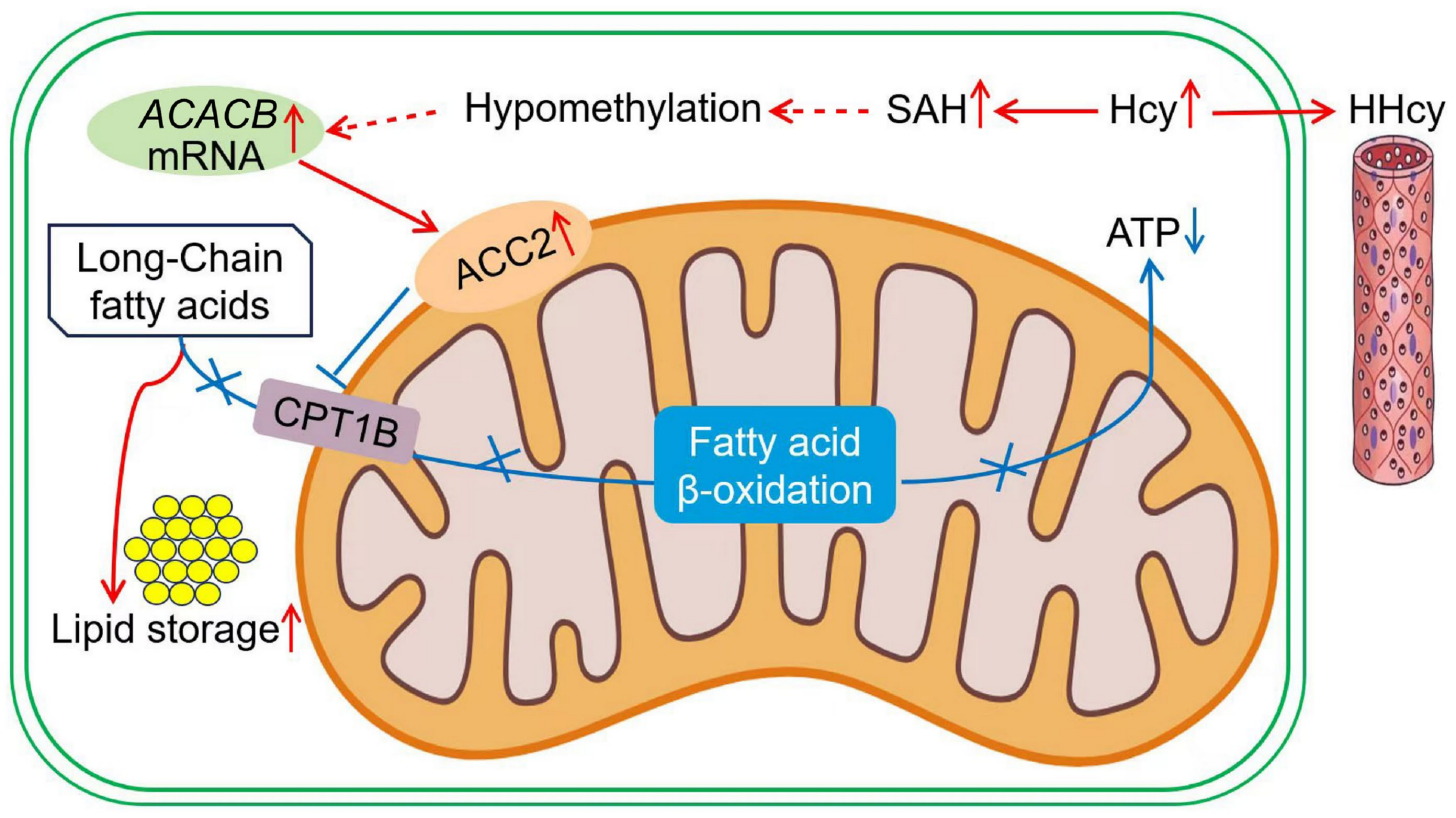
Proposed putative mechanistic pathway underlying the association between HHcy and skeletal muscle lipid deposition. HHcy may trigger the accumulation of S-adenosylhomocysteine (SAH), which is speculated to induce DNA hypomethylation in the promoter region of the acetyl-CoA carboxylase β (*ACACB*) gene, thereby potentially upregulating acetyl-CoA carboxylase 2 (ACC2) expression. The increase in ACC2 enhances the production of malonyl-CoA, a potent allosteric inhibitor of carnitine palmitoyltransferase 1B (CPT1B). Inhibition of CPT1B activity prevents the transport of long-chain fatty acids into the mitochondria for β-oxidation, resulting in reduced adenosine triphosphate (ATP) production and the cytosolic accumulation of triglyceride (TG) as lipid droplets, ultimately leading to lipid deposition.

Lipids, a class of hydrophobic biomolecules, primarily include fatty acids and their derivatives such as TG. Fatty acids serve as the main energy source for muscle during rest and prolonged low-intensity exercise. Within the mitochondrial matrix, fatty acids undergo *β*-oxidation to generate adenosine triphosphate (ATP). Long-chain fatty acids, which are richer ATP sources, enter mitochondria via the CPT system, comprising two separate proteins located in the outer (CPT1) and inner (CPT2) mitochondrial membranes. CPT1 catalyzes the rate-limiting step of fatty acid β-oxidation and is inhibited by malonyl-CoA, the product of ACC2. Our data reveal that HHcy is associated with elevated ACC2 expression, increased malonyl-CoA content and reduced CPT1 activity in skeletal muscle, accompanied by marked accumulation of muscle TG. These correlative molecular changes lead us to propose a hypothetical pathway in which altered ACC2-malonyl-CoA-CPT1 signaling may impair fatty acid oxidation and subsequently promote lipid deposition (Figure 7). Further functional verification experiments, such as *ACACB* knockdown or pharmacological inhibition, are needed to validate this mechanistic hypothesis in future studies.

A notable finding of this study is that Hcy treatment did not affect ACC phosphorylation, suggesting that Hcy regulates ACC2 independently of AMPK-mediated phosphorylation. Nevertheless, the mechanism underlying Hcy-induced upregulation of ACC2 at both mRNA and protein levels remains unclear. We hypothesize that impaired intracellular methylation represents a key contributing factor. Methylation is a fundamental biochemical process involving the transfer of methyl groups from a methyl donor, primarily S-adenosylmethionine (SAM), to substrates such as nucleic acids and proteins, thereby modulating their function (26–28). Following methyl group donation, SAM is converted to S-adenosylhomocysteine (SAH), a potent competitive inhibitor of SAM-dependent methyltransferases. SAH is reversibly hydrolyzed by SAH hydrolase into Hcy and adenosine. Elevated Hcy levels drive the reaction equilibrium toward SAH accumulation, promoting cellular hypomethylation. This hypothesis is supported by yeast studies demonstrating that Hcy-induced lipid deposition is SAH-dependent, positioning methylation inhibition as a pivotal underlying mechanism (28). DNA methylation, a major epigenetic regulatory mechanism, occurs predominantly at cytosine residues within CpG dinucleotides in human genomic DNA and is generally associated with transcriptional repression (26). Given the presence of a CpG island in the promoter region of the *ACACB* gene (29), we hypothesize that HHcy may enhance ACC2 expression by reducing promoter methylation, although this proposition requires further experimental validation (Figure 7).

Several limitations of this study warrant acknowledgment. First, the transcriptomic analysis was performed with only one control and two patient muscle samples. Such a small sample size may affect statistical reliability and carry a risk of false-positive differential genes. We fully recognize this limitation. Therefore, the transcriptomic findings including the altered expression of *ACACB* are only regarded as preliminary exploratory observations, rather than definitive mechanistic evidence. To minimize false-positive interference and confirm its expression pattern, we further validated the expression of *ACACB* using independent qRT-PCR. Second, the overall clinical sample size of this study is relatively small, thus, larger prospective cohort studies are needed to better define its epidemiological and clinical features. Third, the exclusion of hereditary and metabolic myopathies was incomplete. Whole-exome sequencing was only performed in two patients, and the remaining cases lacked systematic genetic screening. Some late-onset lipid metabolic disorders and mitochondrial diseases may present with mild lipid accumulation and fluctuating myopathic symptoms, and normal acylcarnitine profiles cannot fully rule out inherited myopathies such as MADD. Thus, current evidence is insufficient to categorize the observed phenotype purely as an acquired metabolic myopathy. Fourth, the enrolled patients had multiple potential metabolic confounding factors, including vitamin B_12_ deficiency, low-meat diet, anemia and thyroid diseases. We observed significant lipid deposition in HHcy cell models cultured with sufficient vitamin B_12_, preliminarily suggesting that HHcy could independently induce lipid deposition without relying on vitamin B_12_ deficiency. However, the potential interference of other metabolic confounding factors on our results could not be completely excluded. Therefore, this study could only demonstrate the correlation between HHcy and lipid deposition, rather than confirm a definite causal relationship. Fifth, whether ACC2 upregulation is a key mechanism in HHcy-associated skeletal muscle lipid deposition remains to be elucidated. *ACACB* knockdown or knockout models should be employed in future studies. In addition, the upstream molecular mechanisms by which Hcy modulates ACC2 expression, including potential involvement of endoplasmic reticulum stress, epigenetic regulation, or specific transcription factors activation, remain unclear. Finally, this study focused on impaired fatty acid oxidation, while the potential effects of Hcy on lipid uptake and *de novo* lipogenesis in skeletal muscle also deserve further investigation in future research.

## 5 Conclusion

This study observed a clinical association between HHcy and skeletal muscle lipid deposition in six enrolled patients. Our preliminary mechanistic exploration indicated that HHcy may correlate with increased ACC2 expression and subsequent elevation of malonyl-CoA levels, which potentially suppresses CPT1 activity and fatty acid oxidation, thereby facilitating lipid accumulation in skeletal muscle. The *in vitro* experiments further supported this regulatory pattern. These findings suggest that vitamin therapy targeting Hcy reduction may yield clinical benefits to patients with concurrent HHcy and skeletal muscle lipid deposition.

## Data Availability

The raw data supporting the conclusions of this article will be made available by the authors, without undue reservation.
