# Supplementary material for: Hyperhomocysteinemia promotes lipid deposition in skeletal muscle
Source: Front Neurol. 2026 Jun 16;17:1861632. doi: 10.3389/fneur.2026.1861632 (PMC13314467; doi:10.3389/fneur.2026.1861632)
Supplement: Supplementary file 1 [file Table_1.docx]

| Supplementary Table S1 Primer Sequences | |
| --- | --- |
| Name of the Primer | Sequence(5' to 3') |
| Human *ACACB*-F | CAAGGGTTGCGTGAAAGACG |
| Human *ACACB*-R | GAAGTCCTCCGCACTCTCAG |
| Human *CPT1B*-F | GATTGCAGGCGAGAACACGA |
| Human *CPT1B*-R | ATCAGCAATGTCCAGCAGGG |
| Human *β-actin*-F | TGGCACCCAGCACAATGAA |
| Human *β-actin*-R | CTAAGTCATAGTCCGAATAGAAGCA |
| Mouse *Acacb*-F | TCAGTGTCTCCAACCCCGAC |
| Mouse *Acacb*-R | ACGTTGGCAAAGCAGGAGATG |
| Mouse *β-actin*-F | ACGGCCAGGTCATCACTATTG |
| Mouse *β-actin*-R | CAAGAAGGAAGGCTGGAAAAGA |
